# Salivary Calculus

**Published:** 1862-11

**Authors:** L. C. Lane

**Affiliations:** Professor of Physiology in the Medical Department of the University of the Pacific, San Francisco


### SALIVARY CALCULUS.

Reported by L. C. LANE, M.D., Professor of Physiology in the Medical Department of the University of the Pacific, San Francisco.

A few weeks ago, I was consulted by a gentleman in reference to a tumor situated in the soft parts immediately posterior to the symphyris of the maxilla inferia. On examination, I found that the tissues around the orifice of the excretory duct of the sub-lingual gland were much hypertrophied, and elevated in crest-like ridges, similar to a cock's comb, some of which prominences were nearly one-third of an inch in height, and were in a high state of inflammation. The salivary secretion was not interfered with from the duct, but it was continually being poured out in much profusion. On examination of the soft parts beneath the posterior to the chin, indurated enlargement was readily perceived in them. The appearance of the whole was such as to awaken the suspicion that the tumor was of a malignant character, and perhaps might finally require extirpation, in order to effect either a palliative or a permanent cure.

The patient was ordered some simple topical application to the part, together with the use of dilute aqua chlorinata, as a lotion for the mouth, of which the secretions were exceedingly fetid, and ordered him to return in a few days. On his return the next time, there was found to be a small amount of purulent matter escaping from the outlet of the salivary gland. A small probe was introduced into this duct, yet nothing unusual could be felt. The patient returned again in nearly a week afterwards, when I found that an opening had formed in the tumor, more than half an inch behind the exterior to the normal excretory duct of the sub-lingual gland, inside of the mouth and close to the jaw; from this opening, he said that he had "picked out a small stone." The stone proved to be a salivary calculus, weighing nearly an eighth of an ounce.

This concretion was found to be composed of concentric layers of white lime-like substance. Upon pulverizing a small portion of it, and testing with a solution of nitrate of silver, the yellow reaction was shown which characterizes the crystalline phosphates,—showing that it was chiefly composed of the phosphate of lime.

After the discharge of this calculus, the ranula-like enlargement beneath the tongue rapidly subsided, and the man was restored to usual health. On inquiry, I learned that this tumor had been of several months' standing, previous to his applying for any medical assistance; that sometimes it had been much swollen and painful, when the swelling would subside again.—*San Francisco Medical Press.*

